# Cigarette Smoking and Mortality in Japan: The Miyagi Cohort Study

**DOI:** 10.2188/jea.14.S12

**Published:** 2005-03-18

**Authors:** Atsushi Hozawa, Takayoshi Ohkubo, Junko Yamaguchi, Takashi Ugajin, Yayoi Koizumi, Yoshikazu Nishino, Yoshitaka Tsubono, Daisuke Shibuya, Ichiro Tsuji, Akira Fukao, Shigeru Hisamichi

**Affiliations:** 1 Division of Epidemiology, Department of Public Health and Forensic Medicine, Tohoku University Graduate School of Medicine.; 2 Miyagi Cancer Society.; 3 Department of Public Health, Yamagata University School of Medicine.

**Keywords:** cigarette smoking, mortality, population attributable fraction

## Abstract

BACKGROUND: We examined the association between smoking and all-cause mortality among Japanese men and women.

METHODS: In 1990, 18,945 men and 17,107 women in Miyagi Prefecture in rural northern Japan (40-64 year of age) completed a self-administered questionnaire including items on smoking. Cox regression was used to estimate relative risk (RR) of mortality according to smoking categories, with adjustment for age, education, marital status, past history of diseases, drinking, body mass index, walking, and dietary variables. During 11 years of follow-up, 1,209 men and 499 women had died.

RESULTS: Multivariate RRs of all-cause mortality for current smokers as compared with never smokers were 1.71 (95% confidence interval, 1.44-2.03) for men and 1.44 (95% confidence interval, 1.06-1.94) for women. Among men, risk in past smokers who had quit smoking for 15 years or longer was not different from the risk in never smokers (RR, 0.97; 95% confidence interval, 0.68-1.39). Of all deaths, 34% in men and 4% in women were attributable to current or past smoking.

CONCLUSIONS: This study indicates that smoking increases the risk of premature death among middle-aged Japanese men and women and that substantial proportion of death, especially for men, is attributable to smoking.

Cigarette smoking is an established risk factor for all-cause mortality among Caucasians^1^^-^^8^ as well as Japanese.^9^^-^^11^ Since smoking habit often accompanies with unhealthy lifestyle, appropriate control for confounding factors is necessary to estimate the hazard of cigarette smoking.^12^^-^^14^ However, there have been only one Japanese study that analyzed the relative risk (RR) for all-cause mortality associated with cigarette smoking adjusted for possible confounders.^9^ Therefore, we conducted a prospective study among Japanese men and women to examine the association between cigarette smoking and all-cause mortality. The objective of this study was (1) to clarify the association between smoking habit and the other risk factors, (2) to investigate the association between smoking habit and all-cause mortality, (3) to examine the proportion of premature death from all causes attributable to cigarette smoking, and (4) to examine the effect of duration of smoking cessation on all-cause mortality.

## METHODS

### Study Cohort

We have reported the design of this prospective cohort study in detail elsewhere.^15^ Briefly, from June through August 1990, we delivered a self-administered questionnaire on various health habits to 51,921 subjects (25,279 men and 26,642 women) who were 40-64 years of age and lived in 14 municipalities of Miyagi Prefecture in northern Japan. The questionnaires were delivered to and collected from the subjects’ residences by members of health promotion committees appointed by the municipal governments. Usable questionnaires were returned from 47,605 subjects (22,836 men and 24,769 women), yielding a response rate of 91.7%. The study protocol was approved by the institutional review board of Tohoku University Graduate School of Medicine. We considered the return of the self-administered questionnaires signed by the subjects to imply their consent to participate in the study.

### Exposure Data

For the assessment of smoking status, the questionnaire asked firstly if subjects were current, ex-, or never smokers. Current and past smokers were further asked about the age at which they started smoking, and the average number of cigarettes smoked per day. Past smokers were also asked the age they quit smoking. Pack-years were defined as the number of years of smoking * average number of cigarettes per day / 20.

### Follow-up

Of 47,605 subjects who responded to the questionnaire, we excluded 1,522 subjects who indicated that they had prior histories of cancer (n=561), stroke (n=379), or myocardial infarction (n=582). We also excluded 539 subjects who had prevalent cancer, which we ascertained by record linkage to the population-based cancer registry covering the study area.^16^ Furthermore, we excluded 9,492 subjects who provided incomplete information on smoking status, the number of cigarettes smoked per day, age of starting smoking, or years since smoking cessation. Consequently, 36,052 subjects (18,945 men and 17,107 women) with 1,708 deaths (1,209 men and 499 women) remained for this analysis.

We followed up vital and residential status of subjects from June 1, 1990, through March 31, 2001. For this follow-up, we established the Follow-up Committee that was consisted of Miyagi Cancer Society; Community Health Division of all 14 municipalities; Department of Health and Welfare, Miyagi Prefectural Government; and Division of Epidemiology, Tohoku University Graduate School of Medicine. The Committee periodically reviewed the Residential Registration Record of each municipality. With this review, we identified the subjects who either died or emigrated during observation. For both decedents and emigrants, we recorded the date of death or emigration. For decedents, we investigated cause of death by reviewing the death certificates of the subjects at Public Health Centers of the study area. The underlying cause of death was coded according to International Classification of Diseases, the Ninth Revision (ICD9). We discontinued follow-up of subjects who emigrated from the study municipalities because of logistical limitations.

We counted person-years of follow-up for each subject from June 1, 1990, until the date of death, date of emigration outside the study districts, or the end of the study period (March 31, 2001), whichever occurred first. A total of 372,923 person-years accrued. There were 1,740 subjects (4.8% of the analytic cohort) who emigrated from the study municipalities and were lost to follow-up.

### Statistical Analysis

We used Cox proportional-hazards regression to estimate RR and 95% confidence interval (CI) of all-cause mortality according to categories of smoking habit and to adjust for potentially confounding variables, using the PHREG procedure on SAS^®^ version 8.2 statistical software package (SAS Inc., Cary, NC, USA). We conducted all analyses separately for men and women.

We considered the following variables as potential confounders: age in years; body mass index in kg/m^2^ (less than 18.5, 18.5-24.9, or 25 or higher); education (up to 15 years of age, 16-18, or 19 years or older); marital status at baseline (whether or not living with spouse); past histories of hypertension, renal diseases, liver diseases, diabetes mellitus, peptic ulcers, or tuberculosis; alcohol drinking (never drinkers, ex- drinkers, current drinkers); walking time per day (less than 1 hour, or 1 hour or longer); and consumption frequencies of green vegetables and oranges (almost daily, 3-4 times per week, 1-2 times per week, or 1-2 times per month or less often).

We repeated all analyses after excluding the subjects who died during the first three years of follow-up. P values for tests of linear trends were calculated by treating the categories for numbers of cigarette smoking per day or the categories of pack-years as ordinal variables, with the exclusion of ex-drinkers. All P values were two-tailed. Population attributable fraction (PAF) was calculated as pd* {RR-1} / RR where pd = proportion of cases exposed to the risk factor, i.e., the combined proportion of current and past smokers.^17^ This formula is known to be more valid than the popular form (RR-1)*Pe/{1+(RR-1)*Pe}, where Pe = proportion of source population exposed to the risk factor, when confounding variables exist.^17^

## RESULTS

Table 1 shows the baseline characteristics of the 18,945 men and 17,107 women according to smoking status. Compared with never smokers, current smokers were younger, more likely to be current drinkers, less likely to be overweight, and consumed green vegetables and oranges less often. The proportion of subjects with spouse was lower in current smokers than in never smokers among women but not among men. The proportions of the subjects with history of hypertension, liver disease, diabetes, peptic ulcers and tuberculosis were higher in past smokers.

**Table 1.** Characteristics of subjects according to smoking categories.

	Men					Women				
	
	Never smokers	Past smokers	Current smokers (number of cigarettes / day)			Never smokers	Past smokers	Current smokers (number of cigarettes / day)		
	
			All	1 - 19	20≤			All	1 - 19	20≤
No. of subjects	3,922	3,118	11,905	2,984	8,921	15,466	253	1,388	969	419
Mean age (SD)	51.6 (7.1)	52.5 (7.4)	51.1 (7.7)	52.6 (7.9)	50.4 (7.5)	51.5 (7.5)	50.8 (8.2)	50.0 (7.3)	49.8 (7.4)	49.4 (6.9)
Body Mass Index (%)										
<18.5	1.4	1.4	2.4	2.6	2.3	2.6	4.5	4.9	4.4	6.0
18.5-24.9	66.0	65.6	73.9	76.5	73.0	67.0	65.5	67.3	69.4	62.6
25.0≤	32.6	33.0	23.7	20.9	24.6	30.4	30.1	27.8	26.2	31.4
Education (%)										
≤15	40.6	39.7	40.0	41.3	39.5	37.4	44.1	40.4	39.2	43.3
16-18	44.4	43.3	46.4	45.2	46.8	48.4	39.4	48.4	49.2	46.7
19≤	15.0	17.0	13.7	13.5	13.7	14.1	16.5	11.2	11.7	10.0
Living with spouse (%)										
Yes	92.1	94.6	92.3	92.8	92.2	87.9	78.1	81.0	82.1	78.2
No	7.9	5.4	7.7	7.2	7.8	12.1	21.9	19.0	17.9	21.8
Past history (%)										
Hypertension	17.9	23.4	16.1	17.8	15.5	19.1	20.2	16.7	15.7	19.1
Renal diseases	3.6	3.1	2.9	3.9	2.6	4.0	3.6	4.5	4.4	4.5
Liver diseases	5.1	7.5	6.5	6.9	6.3	3.5	4.4	4.0	3.5	5.0
Diabetes mellitus	4.9	5.7	5.1	5.2	5.0	2.8	4.4	2.7	2.7	2.6
Peptic ulcers	14.5	23.3	21.8	20.2	22.4	9.1	20.2	13.3	12.8	14.3
Tuberoculosis	3.3	4.0	2.8	2.9	2.8	2.4	4.7	2.9	2.9	2.9
Green vegetables (%)										
≤1-2 times/month	12.8	13.1	16.3	14.5	16.9	7.6	8.5	14.9	13.5	18.3
1-2 times/week	34.4	33.4	36.0	34.0	36.7	29.1	29.7	34.2	33.7	35.5
3-4 times/week	31.3	31.4	28.6	29.9	28.2	36.3	35.2	31.7	32.8	29.0
Everyday	21.6	22.1	19.1	21.6	18.2	27.0	26.7	19.2	20.0	17.2
Oranges (%)										
≤1-2 times/month	23.1	25.3	32.6	30.6	33.2	14.2	18.7	24.8	22.5	30.4
1-2 times/week	30.7	29.3	29.9	28.0	30.5	20.0	21.3	23.3	23.7	22.4
3-4 times/week	24.8	25.6	21.5	23.0	21.1	27.4	28.1	25.4	26.2	23.5
Everyday	21.4	19.8	16.0	18.5	15.2	38.5	31.9	26.5	27.7	23.8
Alcohol drinking (%)										
Never	22.8	12.1	14.7	13.5	15.0	76.5	36.3	34.6	35.3	32.8
Past	5.0	10.8	6.3	7.2	6.0	2.4	21.5	13.0	12.0	15.4
Current	72.3	77.1	79.0	79.3	79.0	21.1	42.2	52.4	52.7	51.8
Walking (%)										
1 hr/day≤	46.5	41.6	45.7	47.3	45.2	44.2	42.6	42.1	43.9	37.8
<1 hr/day	53.5	58.4	54.3	52.7	54.8	55.8	57.4	57.9	56.1	62.2

SD denotes standard deviation.

Table 2 presents RR for all-cause mortality according to smoking categories. For both sex, age-adjusted analysis showed that the risk of death was significantly higher in current smokers than in never smokers. The risk was also significantly higher in past smokers than in never smokers, although point estimates of RR for past smokers were lower than those for current smokers. These results remained basically unchanged after multivariate adjustment or after the exclusion of subjects who died during the first three years of follow-up.

**Table 2.** Relative risk (RR) for all cause mortality according to smoking status in men and women.^†^

	Never smokers	Past smokers	Current			P for trend

			All	1-19/day	20/day≤	
Men						
No. of subjects	3,922	3,118	11,905	2,984	8,921	
Person-years	40,766	31,955	121,999	30,588	91,411	
No. of death	163	206	840	220	620	
Age-adjusted RR	1.0	1.43 (1.17 - 1.76)	1.79 (1.51 - 2.11)	1.59 (1.30 - 1.95)	1.87 (1.57 - 2.22)	<0.001
Multivariate RR1	1.0	1.38 (1.12 - 1.70)	1.71 (1.44 - 2.03)	1.50 (1.22 - 1.84)	1.80 (1.51 - 2.14)	<0.001
Multivariate RR2	1.0	1.33 (1.06 - 1.66)	1.72 (1.44 - 2.07)	1.52 (1.22 - 1.89)	1.81 (1.50 - 2.19)	<0.001
Women						
No. of subjects	15,466	253	1,388	969	419	
Person-years	161,572	2,565	14,066	9,891	4,175	
No. of death	436	9	54	38	16	
Age-adjusted RR	1.0	1.26 (0.65 - 2.44)	1.64 (1.24 - 2.18)	1.62 (1.16 - 2.25)	1.70 (1.03 - 2.81)	0.001
Multivariate RR1	1.0	1.10 (0.56 - 2.16)	1.44 (1.06 - 1.94)	1.47 (1.04 - 2.08)	1.35 (0.81 - 2.26)	0.031
Multivariate RR2	1.0	1.34 (0.68 - 2.65)	1.42 (1.02 - 1.98)	1.40 (0.95 - 2.06)	1.47 (0.85 - 2.55)	0.037

† : Adjusted for age in years; body mass index in kg/m^2^ (less than 18.5, 18.5-24.9, or 25 or higher); education (up to 15 years of age, from 16-18, or 19 years or older); marital status at baseline (whether or not living with spouse); past histories of hypertension, renal diseases, liver diseases, diabetes mellitus, peptic ulcers, or tuberculosis; alcohol drinking (never drinkers, past drinkers, current drinkers); walking time per day (less than 1 hour, or 1 hour or longer); consumption frequencies of green vegetables and oranges (almost daily, 3-4 times per week, 1-2 times per week, or 1-2 times per month or less often). Multivariate RR2 has been estimated with the exclusion of 264 subjects (179 men and 85 women) who died within the first 3 years of follow-up . Numbers in parentheses are 95% confidence intervals.

Tables 2 and 3 present RR and 95% CIs for all-cause mortality by the number of cigarette smoked per day and by pack-years of smoking, respectively. For men, age-adjusted analysis showed that statistically significant linear trend of RR was seen for both the number of cigarette smoked per day and for pack-years. These results remained basically unchanged after multivariate adjustment or after the exclusion of subjects who died during the first three years of follow-up. For women, the linear trends of RR were less apparent than those observed among men. Based on the multivariate RR (95%CI) for current and past smokers combined as compared with never smokers [1.63 (1.38-1.93) for men and 1.38 (1.04-1.83) for women], PAFs of smoking were 34% for men and 4% for women.

**Table 3.** Relative risk (RR) for all-cause mortality according to pack-years of smoking in men and women.^†^

	Pack-years of Smoking				P for trend
Men	Never	1-19	20-39	40≤	
Np. of subjects	3,922	3,927	7,173	3,923	
Person-Years	40,766	40,481	73,699	39,774	
No. of death	163	198	492	356	
Age-adjusted RR	1.0	1.49 (1.21 - 1.84)	1.72 (1.44 - 2.05)	1.83 (1.52 - 2.20)	<0.001
Multivariate RR1	1.0	1.41 (1.14 - 1.74)	1.65 (1.38 - 1.98)	1.76 (1.46 - 2.13)	<0.001
Multivariate RR2	1.0	1.35 (1.08 - 1.70)	1.71 (1.41 - 2.07)	1.71 (1.40 - 2.10)	<0.001

Women	Never	1-19	20-		
Np. of subjects	15,466	1,320	321		
Person-Years	161,572	13,468	3,163		
No. of death	436	47	16		
Age-adjusted RR	1.0	1.53 (1.13 - 2.06)	1.72 (1.05 - 2.83)		<0.001
Multivariate RR1	1.0	1.39 (1.01 - 1.90)	1.35 (0.80 - 2.26)		0.044
Multivariate RR2	1.0	1.42 (1.01 - 2.00)	1.37 (0.77 - 2.44)		0.049

†: Adjusted for age in years; body mass index in kg/m^2^ (less than 18.5, 18.5-24.9, or 25 or higher); education (up to 15 years of age, from 16-18, or 19 years or older); marital status at baseline (whether or not living with spouse); past histories of hypertension, renal diseases, liver diseases, diabetes mellitus, peptic ulcers, or tuberculosis; alcohol drinking (never drinkers, past drinkers, current drinkers); walking time per day (less than 1 hour, or 1 hour or longer); consumption frequencies of green vegetables and oranges (almost daily, 3-4 times per week, 1-2 times per week, or 1-2 times per month or less often). Multivariate RR2 has been estimated with the exclusion of 264 subjects (179 men and 85 women) who died within the first 3 years of follow-up . Numbers in parentheses are 95% confidence intervals.

Table 4 presents the change in the multivariate RRs of all-cause mortality for male past smokers with different durations of smoking cessation. For men, the risk of death for past smokers who had quit for 15 years or shorter was significantly higher as compared with never smokers. In contrast, the RR for subjects who had quit smoking for 15 years or longer was not significantly higher as compared with never smokers. Since we observed only 9 deaths among female past smokers, we did not conduct similar analysis.

**Table 4.** Multivariate relative risk (RR) for all-cause mortality by years since quitting among past smokers in men.^†^

	Current smokers	Past smokers, by years since quitting				Never smokers

		1-4	5-9	10-14	15≤	
No. of subjects	11,905	965	827	653	673	3,922
Person-Years	121,999	9,850	8,504	6,595	7,007	40,766
No. of death	840	71	52	45	38	163
Multivariate RR	1.71 (1.44 - 2.03)	1.50 (1.14 - 1.99)	1.46 (1.07 - 2.00)	1.63 (1.17 - 2.27)	0.97 (0.68 - 1.39)	1.00

† : Adjusted for age in years; body mass index in kg/m^2^ (less than 18.5, 18.5-24.9, or 25 or higher); education (up to 15 years of age, from 16-18, or 19 years or older); marital status at baseline (whether or not living with spouse); past histories of hypertension, renal diseases, liver diseases, diabetes mellitus, peptic ulcers, or tuberculosis; alcohol drinking (never drinkers, past drinkers, current drinkers); walking time per day (less than 1 hour, or 1 hour or longer); consumption frequencies of green vegetables and oranges (almost daily, 3-4 times per week, 1-2 times per week, or 1-2 times per month or less often). Numbers in parentheses are 95% confidence intervals.

## DISCUSSION

This prospective cohort study presented the association between cigarette smoking and all-cause mortality in the Japanese general population. We demonstrated that (1) smoking habit was related to other unhealthy habits; (2) mortality in current smokers was higher than that in never smokers; (3) 34% and 4% of premature death are attributable to cigarette smoking in men and in women, respectively; (4) the risk in past smokers was higher than never smokers but lower than that in current smokers; and (5) among men, risk in past smokers who stopped smoking for 15 years or longer was not different from the risk in never smokers.

In this study, compared with never smokers, current smokers were younger, less likely to be overweight, never drinkers, and consume green vegetables and oranges. Proportions of complication were higher in past smokers. The proportion of the subjects with spouse was lower in current smokers. These findings, especially for dietary patterns, were consistent with previous studies. Kaetsu et al. reported that the vegetable or fruits intake was lower in smokers than in non-smokers.^12^ Similarly, Bottoni et al. reported that smokers consumed foods that were less varied than non-smokers.^13^ Smokers were also reported to eat vegetables or fruit less frequently and consume more alcohol than non-smokers.^13^ These findings point to the necessity of adjustment for life-style variables or complications in examining the association between smoking and mortality.

In Japan, some studies reported the risk of smoking habit on all-cause mortality.^9^^-^^11^ All of them demonstrated that smoking habit was harmful. However, there has been only one study in Japan that analyzed the RR for all-cause mortality associated with cigarette smoking with extensive adjustment for possible confounders.^9^ In that study, Hara et al. followed up 19,950 men and 21,534 women aged 40-59 for 10 years and ascertained 1,014 deaths among men and 500 among women. After adjustment for age, area, educational background, history of hypertension, any medication, sports in leisure time, consumption frequencies of several foods (green vegetables, yellow vegetables, white vegetables, fruit, fish, pickled vegetables, and soy products), they reported that RR (95% CI) for current smokers as compared with never smokers was 1.55 (1.29-1.86) for men and 1.89 (1.36-2.62) for women. The authors discussed that the RR of current smokers for all-cause mortality was lower than that in western populations. Considering that these values were consistent with our corresponding RRs (1.71 for men and 1.44 for women), the RR of current smokers for all-cause mortality in Japanese population may be lower than that in western populations. Hara et al. discussed that the discrepancy between the RR of current smokers for all-cause mortality in Japanese population and that in western population might be explained by the difference in exposure dose or race. However, in our study, even the highest cumulative dose group did not exceed the 2 fold increase in risk compared with non-smokers in both sexes. These tendencies were also found in the report by Hara et al, except for the highest cumulative dose group in women. Therefore, we considered that the difference might be attributed by racial difference in genetic and other lifestyle factors.

Hara et al. also reported that population attributable fractions of mortality by smoking were 22% for men and 5% for women, and their estimates were consistent with ours (34% for men and 4% for women). The two studies therefore suggest that the 20-30% for men and 5% for women of premature death from all-causes are attributable to cigarette smoking in the Japanese population.

We found that past smokers had risk of death that was higher than never smokers but lower than current smokers. We also observed among men that past smokers who had quit smoking for shorter than 15 years had significantly higher mortality than never smokers, while past smokers who had quit smoking for 15 years or longer had risk of death that was comparable to never smokers. In another Japanese prospective study examining the association between duration of smoking cessation and all-cause mortality,^11^ risk of death among male past smokers decreased to the level of nonsmokers with 10 or longer years of cessation, while no such decrease in risk was observed for female past smokers. The U.S. Surgeon General’s Report on the Health Benefits of Smoking Cessation concluded that (1) among former smokers, the decline in risk of death compared with continuing smokers begins shortly after quitting and continues for at least 10 to 15 years, and (2) after 10 to 15 years of abstinence, risk of all-cause mortality returns nearly to that of persons who never smoked.^18^ Our results are consistent with these conclusions.

The strengths of our study are as follows. First, our subjects represent the general population of study area and we achieved high follow-up rate. Second, we adjusted possible confounders such as physical activity, alcohol drinking, dietary intake, socioeconomic status, body mass index, and past history of diseases. Third, we observed similar findings when we excluded from analyses subjects who died during earlier years of follow-up in order to minimize the influence of life style changes due to ill health that had already been present at baseline. Therefore, we could consider our results as having a high external and internal validity.

In conclusion, this study indicates that smoking increased the risk of premature death among middle-aged Japanese men and women and that substantial proportion of death, especially for men, is attributable to smoking.
